# Soil Moisture and Soluble Salt Content Dominate Changes in Foliar δ^13^C and δ^15^N of Desert Communities in the Qaidam Basin, Qinghai-Tibetan Plateau

**DOI:** 10.3389/fpls.2021.675817

**Published:** 2021-07-08

**Authors:** Weiling Niu, Hui Chen, Jianshuang Wu

**Affiliations:** ^1^Hebei Key Laboratory of Environmental Change and Ecological Construction, College of Resources and Environmental Sciences, Hebei Normal University, Shijiazhuang, China; ^2^Hebei Technology Innovation Center for Remote Sensing Identification of Environmental Change, College of Resources and Environmental Sciences, Hebei Normal University, Shijiazhuang, China; ^3^Institute of Environment and Sustainable Development in Agriculture, Chinese Academy of Agricultural Sciences, Beijing, China; ^4^Theoretical Ecology, Institute of Biology, Freie Universität Berlin, Berlin, Germany

**Keywords:** carbon isotopic composition, environmental gradients, nitrogen isotopic composition, nitrogen utilization strategy, water use efficiency

## Abstract

Changing precipitation and temperature are principal drivers for nutrient cycling dynamics in drylands. Foliar isotopic carbon (C) and nitrogen (N) composition (δ^13^C and δ^15^N) are often used to describe the plant’s water use efficiency and nitrogen use strategy in plant ecology research. However, the drivers and mechanisms under differential foliar δ^13^C and δ^15^N among plant species and communities are largely unknown for arid high-elevation regions. This study collected 462 leaf samples of ten top-dominant plant species (two or three replicates per species) across 16 sites in 2005 and 2010 to measure the community-weighted means (CWMs) of foliar δ^13^C and δ^15^N, northeastern Qaidam Basin, Qinghai-Tibetan Plateau. Our results showed that the CWM of foliar δ^15^N was higher in 2005 than in 2010 and was lower in the warm-dry season (July and August) than the cool-wet one (June and September) in 2010. Similarly, the CWM of foliar δ^13^C was higher in 2005 than in 2010, but no difference between warm-dry and cool-wet seasons in 2010. C_4_ plants have higher δ^13^C and generally grow faster than C_3_ species under warm-wet weathers. This might be why the CWM of foliar δ^13^C was high, while the CWM of foliar δ^15^N was low in the wet sampling year (2010). The general linear mixed models revealed that soil moisture was the most critical driver for the CWM of foliar δ^15^N, which explained 42.1% of the variance alone. However, the total soluble salt content was the crucial factor for the CWM of foliar δ^13^C, being responsible for 29.7% of the variance. Growing season temperature (GST) was the second most vital factor and explained 28.0% and 21.9% of the variance in the CWMs of foliar δ^15^N and δ^13^C. Meanwhile, remarkable differences in the CWMs of foliar δ^15^N and δ^13^C were also found at the species level. Specifically, *Kalidium gracile* and *Salsola abrotanoides* have higher foliar δ^15^N, while *Ephedra sinica* and *Tamarix chinensis* have lower foliar δ^15^N than other species. The foliar δ^13^C of *Calligonum Kozlov and H. ammodendron* was the highest among the ten species. Except for the foliar δ^13^C of *E. sinica* was higher than *Ceratoide latens* between the two sampling years or between the cool-wet and warm-dry seasons, no significant difference in foliar δ^13^C was found for other species. Overall, the CWMs of foliar δ^15^N and δ^13^C dynamics were affected by soil properties, wet-dry climate change, and species identity in high-elevation deserts on the Qinghai Tibetan Plateau.

## Introduction

Isotopic carbon (C) and nitrogen (N) composition (δ^13^C and δ^15^N) can provide fundamental insights into ecosystem biogeochemical cycles (Handley et al., 1999). For example, foliar δ^13^C can infer intrinsic water use efficiency (WUE) of C_3_ plants (Hultine and Marshall, 2000; Warren et al., 2001; Qiang et al., 2003) while δ^15^N can reveal nitrogen use efficiency (NUE) and fractionation during the N-uptake, transport, transform, and decomposition (Robinson, 2001). Therefore, both δ^13^C and δ^15^N can be used to explore how plants respond to environmental changes (Adams and Grierson, 2001; Canadell et al., 2002; Dawson et al., 2002).

The δ^13^C of terrestrial plant fossils is also increasingly used to reconstruct paleoclimate (Siegwolf, 2007; Werner et al., 2012) because the δ^13^C of plant issues has recorded a series of climate change information associated with plant growth (Wang et al., 2005; Chen et al., 2007; Wang et al., 2010; Ma et al., 2012). Therefore, the δ^13^C can also serve as a valuable indicator of plant physiology (Saurer et al., 1995; Loader et al., 2007; Dodd et al., 2008; Diefendorf et al., 2010). Precipitation and temperature are the essential factors for plant growth, fitness, and performance in drylands (Huxman et al., 2004; Newman et al., 2006). Plant δ^13^C declines with increasing mean annual precipitation (MAP), likely due to the intrinsic WUE among species (Golluscio and Oesterheld, 2007; Moreno-Gutierrez et al., 2012). For example, Song et al. (2008) reported that foliar δ^13^C of dominant plants could describe alpine species differentiation in response to water availability across the Tibetan Plateau. However, it is still under debates about how foliar δ^13^C varies with mean annual temperature (MAT). Foliar δ^13^C has been reported to be positively (Li et al., 2005; Wang and Schjoerring, 2012; Wang, 2018), negatively correlated with MAT (Song et al., 2008; Zhou et al., 2011), and even no clear relation between them (Li et al., 2009). However, Yang et al. (2015) found that soil properties could explain more variance in δ^13^C than climatic factors at high-elevation grasslands. They even found a unimodal pattern between foliar δ^13^C and soil organic carbon (SOC) for alpine steppes on the Tibetan Plateau. Besides, high N availability in soils could lead to high foliar δ^13^C, primarily due to structural changes in plant tissue under droughts (Bol et al., 2004).

Similarly, foliar and soil δ^15^N decreases with MAP in drylands (Handley et al., 1999; Aranibar et al., 2004), implying ecosystem N cycling might be more open (Robinson, 2001). Martinelli et al. (1999) found that foliar δ^15^N was higher in tropical forests than temperate ones, indicating that plant NUE may increase with increasing temperature. In addition to temperature and precipitation, soil properties are also essential in controlling ecosystem N cycling (Booth et al., 2005; Chapin et al., 2011). For example, soil moisture can promote N mineralization and nitrification by affecting microbial activity (Butterbach-Bahl and Gundersen, 2011; Chapin et al., 2011). Soil pH can affect microbial nitrification and denitrification, NH_3_ volatilization (Booth et al., 2005; Butterbach-Bahl and Gundersen, 2011; Chapin et al., 2011). Soil texture and mycorrhizal fungi can also significantly influence vegetation δ^15^N via plants’ N-uptake preference and fractionation during the N-transfer between plant and mycorrhiza (Beyschlag et al., 2009; Klaus et al., 2013). Therefore, soil properties are also expected to affect vegetation δ^15^N variation, especially at high-elevation drylands.

Plant δ^13^C and δ^15^N in response to changes in temperature and precipitation are species-specifically different (Robinson, 2001; Golluscio and Oesterheld, 2007; Lazaro-Nogal et al., 2013). In addition to habitat conditions, species physiological traits can also regulate the variability of foliar δ^15^N and δ^13^C (Elmore et al., 2017). For example, Gatica et al. (2017) found that the short-term interaction between environmental change and plant functional traits may override temperature to affect plant δ^13^C and δ^15^N in drylands. Current findings on the trait-regulating effects on foliar δ^15^N and δ^13^C are mainly from temperate (Garten et al., 2000; Peri et al., 2012) and tropical regions (Powers and Schlesinger, 2002) but less from alpine biomes. Hgh-elevation deserts are sensitive to climate warming and wetting (Yang et al., 2009; Lin et al., 2011; Lu et al., 2013). Warming-induced C and N losses from alpine soils can even offset C and N sequestration by vegetation, triggering positive feedback to climate warming (Tan et al., 2010; Lu et al., 2013). Therefore, a better understanding of the mechanisms governing C- and N- related processes is crucial in high-elevation ecosystems (Yang et al., 2009; Averill et al., 2014).

This study explored how climate change (warm-wet vs cool-dry) affects foliar δ^13^C and δ^15^N of alpine desert plants in the northeastern Qaidam Basin, Qinghai-Tibetan Plateau. We hypothesized that changes in precipitation and temperature affect plant δ^13^C and δ^15^N differently among species genotypes. Specifically, we aim to (1) examine the differences in foliar δ^13^C and δ^15^N under the dry-wet change; (2) explore how foliar δ^13^C and δ^15^N respond to changes in climate and soil factors; and (3) to evaluate the relative contribution of environmental factors to changes in foliar δ^13^C and δ^15^N at both plant species and community levels.

## Materials and Methods

### Study Area

**FIGURE 1:**
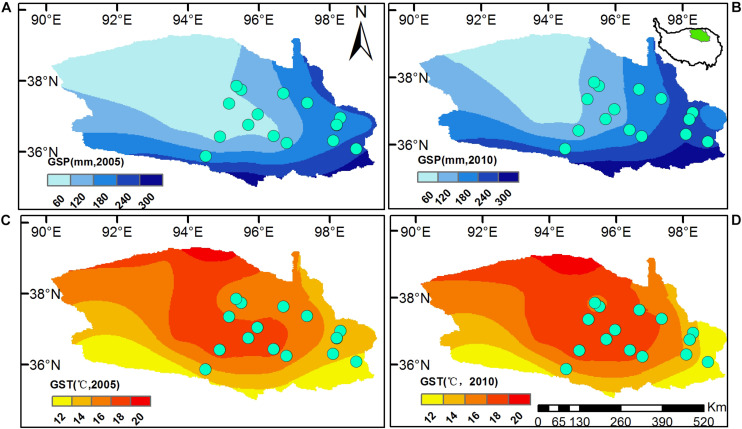
Locations of sampling sites in the Eastern Qaidam Basin, northeastern Qinghai-Tibetan Plateau. Raster surfaces are the sum precipitation [GSP, **(A,B)**] and mean temperature [GST, **(C,D)**] during the plant growing seasons, from May to September 2005 and 2010. Climatic surfaces were generated using ArcGIS 10.2, with daily weather records downloaded from the China Meteorological Data Service Center (CMDC, http://data.cma.cn/en). The small map embedded in **(B)** shows the Qaidam Basin’s location in the Qinghai-Tibetan Plateau.

The study area locates in the East Qaidam Basin, Qinghai-Tibetan Plateau (Figure 1), with evident differences in climate (Figure 1), soil nutrients and plant assembly (Tables 1, 2). In this study, we had 11 sites sampled in 2005 and five sites in 2010. The site elevation is between 2500 m and 3600 m (Table 1). Mean temperature and sum precipitation during the plant growing season (from May to September, GST and GSP) range from 10 to 17.4°C and from 35.9 to 224 mm, respectively (Table 1). Soil is arid and salty, with the habitat aridity index ranging from 2.1 to 30.4 mm °C**^–^**^1^ and soil soluble salts from 155 to 2787 EC25 μs cm^–1^. Vegetation is dominated by shrub and semi-shrub halophytes, resistant to drought and salinization (Table 1).

**TABLE 1:** Sampling year, site locations, soil properties, and climate regimes in the Qaidam Basin, northeastern Qinghai-Tibetan Plateau.

Sites	Year	Long	Lat	Alt	SOC	STN	SM	BD	pH	SS	GSP	GST	Aridity
		(°E)	(°N)	(m)	(g kg^–1^)	(g kg^–1^)	(%)	(g cm^–3^)		(EC25 μs cm^–1^)	(mm)	(°C)	(mm °C^–1^)
Golmud-1	2005	94.501	35.866	3620	1.27	0.12	3.20	1.55	8.28	262.47	146.88	11.97	21.21
Golmud-2	2005	95.702	36.752	2842	1.10	0.18	3.10	1.46	8.57	1046.66	46.76	16.80	21.52
Golmud-3	2005	96.786	36.239	2827	1.41	0.48	3.65	1.52	9.05	1868.07	104.17	16.37	15.03
Dulan-1	2005	98.307	36.949	3302	2.49	0.19	10.07	1.36	9.40	302.71	167.90	11.79	2.9
Delhi-1	2005	96.699	37.637	2873	2.34	0.35	3.39	1.67	9.03	567.28	109.17	15.89	9.31
Da Qaidam-1	2005	95.967	37.042	3327	2.64	0.25	4.02	1.63	8.10	815.06	54.25	13.76	17.48
Da Qaidam-2	2005	95.165	37.352	3180	1.43	0.47	7.62	1.64	7.89	910.87	38.94	14.72	21.51
Da Qaidam-3	2005	95.506	37.737	3041	1.84	0.44	0.70	1.76	7.87	536.69	68.59	15.38	11.8
Ulan	2005	98.203	36.740	2969	2.98	1.12	9.44	1.37	8.45	1544.26	161.59	14.01	3.33
Delhi-2	2005	98.203	36.749	2850	2.22	0.38	4.89	1.54	8.93	903.52	161.59	14.73	3.34
Chaka	2005	98.754	36.078	3597	4.37	0.30	12.19	1.54	8.58	1642.37	214.65	9.99	2.11
Da Qaidam-4	2010	95.366	37.850	3173	12.38	0.29	2.63	1.76	7.87	536.69	93.10	15.40	7.47
Delhi-3	2010	97.367	37.367	2982	5.35	0.38	2.22	1.67	8.52	1107.95	169.90	15.99	3.89
Dulan-2	2010	98.100	36.300	3191	3.30	0.50	9.92	1.37	8.64	155.06	237.30	13.86	3.49
Golmud-4	2010	94.900	36.417	2808	1.48	0.09	2.73	1.55	8.10	2787.09	69.50	17.36	13.11
Nuomuhong	2010	96.417	36.433	2790	2.20	0.12	1.74	1.52	9.05	1868.07	128.00	17.19	7.92

*Soil variables include total soil nitrogen (STN), soil organic carbon (SOC), soil moisture (SM), bulk density (BD), pH and soil soluble salts (SS) at the 0–30 cm layer. Climatic factors are the sum precipitation and average temperature during the plant growing season (GSP and GST, respectively) and the habitat aridity index (Aridity). The habitat aridity index is defined as the ratio of PE/MAP, where PE is the potential evapotranspiration calculated with the Penman-Monteith formula.*

**TABLE 2:** Plant cover, nitrogen isotopic composition (δ^15^N, ‰) and carbon isotopic composition (δ^13^C, ‰) of species measured at each site.

Site	Year	Species–cover (%)					Species–δ^15^N (‰)					Species–δ^13^C (‰)				
Golmud-1	2005	*C.l.*	*S.a.*				*C.l.*	*S.a.*				*C.l.*	*S.a.*			
		80	20				11.31	12.00				−24.75	−24.76			
Golmud-2	2005	*T.c*	*C.k.*				*T.c*	*C.k.*				*T.c*	*C.k.*			
		98	2				10.42	15.90				−25.48	−13.13			
Golmud-3	2005	*S.r.*	*H.a.*				*S.r.*	*H.a.*				*S.r.*	*H.a.*			
		10	90				9.37	11.57				−23.04	−15.11			
Dulan-1	2005	*R.s.*	*S.a.*				*R.s.*	*S.a.*				*R.s.*	*S.a.*			
		3	97				13.59	18.82				−27.54	−27.43			
Delhi-1	2005	*S.r.*					*S.r.*					*S.r.*				
		100					9.51					−24.09				
Da Qaidam-1	2005	*C.l.*	*S.r.*	*S.a.*			*C.l.*	*S.r.*	*S.a.*			*C.l.*	*S.r.*	*S.a.*		
							7.81	9.02	10.84			−24.92	−23.65	−22.92		
		90	5	5												
Da Qaidam-2	2005	*C.l.*	*S.a.*				*C.l.*	*S.a.*				*C.l.*	*S.a.*			
		63	37				12.02	14.61				−24.67	−23.21			
Da Qaidam-3	2005	*S.r.*	*E.s.*				*S.r.*	*E.s.*				*S.r.*	*E.s.*			
		24	76				7.65	7.91				−23.81	−22.22			
Ulan	2005	*S.a.*					*S.a.*					*S.a.*				
		1					9.51					−24.09				
Delhi-2	2005	*K.g.*	*S.a.*	*C.l.*			*K.g.*	*S.a.*	*C.l.*			*K.g.*	*S.a.*	*C.l.*		
												−26.60	−23.67	−25.54		
		19	15	66			10.52	15.53	11.14							
Chaka	2005	*S.a.*	*C.l.*				*S.a.*	*C.l.*				*S.a.*	*C.l.*			
		46	54				14.24	13.34				−25.73	−25.82			
Da Qaidam-4	2010	*S.a.*	*C.l.*	*S.r.*			*S.a.*	*C.l.*	*S.r.*			*S.a.*	*C.l.*	*S.r.*		
		32	37	31			8.63	9.15	8.27			−26.77	−28.40	−26.51		
Delhi-3	2010	*S.r.*	*K.g.*				*S.r.*	*K.g.*				*S.r.*	*K.g.*			
		25	75				8.04	6.44				−25.28	−22.79			
Dulan-2	2010	*K.g.*	*S.a.*				*K.g.*	*S.a.*				*K.g.*	*S.a.*			
		95	5				12.88	11.94				−26.90	−27.86			
Golmud-4	2010	*C.l.*	*S.r.*	*E.s.*	*C.k.*		*C.l.*	*S.r.*	*E.s.*	*C.k.*		*C.l.*	*S.r.*	*E.s.*	*C.k.*	
		18	30	12	40		4.98	4.14	1.29	4.23		−27.49	−27.48	−24.19	−13.06	
Nuomuhong	2010	*C.l.*	*N.t.*	*E.s.*	*T.c.*	*C.k.*	*C.l.*	*N.t.*	*E.s.*	*T.c.*	*C.k.*	*C.l.*	*N.t.*	*E.s.*	*T.c.*	*C.k.*
		10	15	35	10	30	5.95	3.98	3.35	3.06	5.36	−27.07	−26.27	−24.93	−25.70	−12.46

*C.l., Ceratoides latens; C.k., Calligogum kozlovi (C_4_); E.s., Ephedra sinica; H.a., Haloxylon ammodendron (C_4_); K.g., Kalidium gracile; N.t., Nitraria tangutorum; R.s., Reaumuria soongorica; S.a., Salsola abrotanoides; S.r., Sympegma regelii; T.c., Tamarix chinensis.*

### Field Surveys in 2005 and 2010

We collected 66 leaf samples of nine dominant species from 11 sites during the peak plant growing season of 2005, from late July and early August. In 2010, 396 leaf samples of eight dominant species at five sites were collected every 2 weeks from June to September. Totally, 462 foliar samples of ten typical desert plants (see details in Table 2 and Supplementary Table 1) were used for further analysis (Figure 1).

First, we chose an open flat area at each site where soil and vegetation were homogeneous without human disturbance and livestock grazing. Five quadrats of 5 m × 5 m were randomly laid to sample short plants at each site and five quadrats of 10 m × 10 m for high ones. We took leaves from two or five dominant species at each plot. For each species, three to five leaves were collected from healthy adult individuals. Leaf samples of the same species were mixed by site, washed with deionized water, and oven-dried at 75°C for 48 h to constant weight in the lab. Finally, leaves were ground into fine powders and stored in glasswares before isotopic analysis.

Second, we collected soil samples at three depths (0–10 cm, 10–20 cm, and 20–30 cm), three soil cores per layer, at each quadrat. Fresh soil samples were first sieved through a 2.0-mm sieve to remove roots, gravels, and stones. Then, we divided each soil sample into two parts: one oven-dried for 24 h at 105°C for soil moisture measurement and the other air-dried for physical and chemical analyses. Soil moisture content was measured as the weight difference between fresh and oven-dried soils.

### Chemical and Isotopic Analyses

Soil total nitrogen (STN, g kg^–1^) was analyzed with the Kjeldahl method of nitrogen determination and soil organic carbon (SOC, g kg^–1^) with the vitriol acid-potassium dichromate oxidation method. Soil pH was measured by a pH electrode in a mixture of soil and water, with a soil: water ratio of 1:2.5. The electrical conductivity or resistivity was used to measure soluble soil salts.

The natural abundance composition of ^15^N/^14^N and ^13^C/^12^C were measured with a stable isotope mass spectrometer (Finnegan Mat-253). The standard error of repeated measurements was ± 0.2‰. The ^15^N/^14^N and ^13^C/^12^C abundance ratio of samples (*R*_sample_) was given in δ notation and expressed in parts per mil (‰) relative to the standard as follows:

(1) $$\delta^{15}N\left(‰\right)=\left(R_{sample}/R_{std}-1\right)*1000$$

(2) $$\delta^{13}C\left(‰\right)=\left(R_{sample}/R_{std}-1\right)*1000$$

where *R*_sample_ and *R*_std_ are the ratios of ^13^C/^12^C or ^15^N/^14^N of the sample and standard, respectively. The Pee Dee Belemnite (PDB) (δ^13^C = 0.0112372) and atmospheric nitrogen (δ^15^N_air_ = 0) were used as international standards for stable carbon and nitrogen, respectively.

In this study, the community-weighted means (CWMs) (CWM) of foliar δ^15^N and δ^13^C were calculated for each site as follow,

(3) $$CWM_{j}=\sum_{i=1}^{n}P_{ij}T_{ij}$$

where the *P*_ij_ is the relative dominance (cover percent) of the species i in the site j; *T*_ij_ is the mean trait value of the species i in the site j; and the CWM_*j*_ is the community weighted mean of the trait at the site j. We also calculated the CWMs of foliar δ^15^N and δ^13^C with and without C_4_ plants to examine the C_4_ plants’ contribution under changing weather conditions.

### Climate Data Processing

**FIGURE 2:**
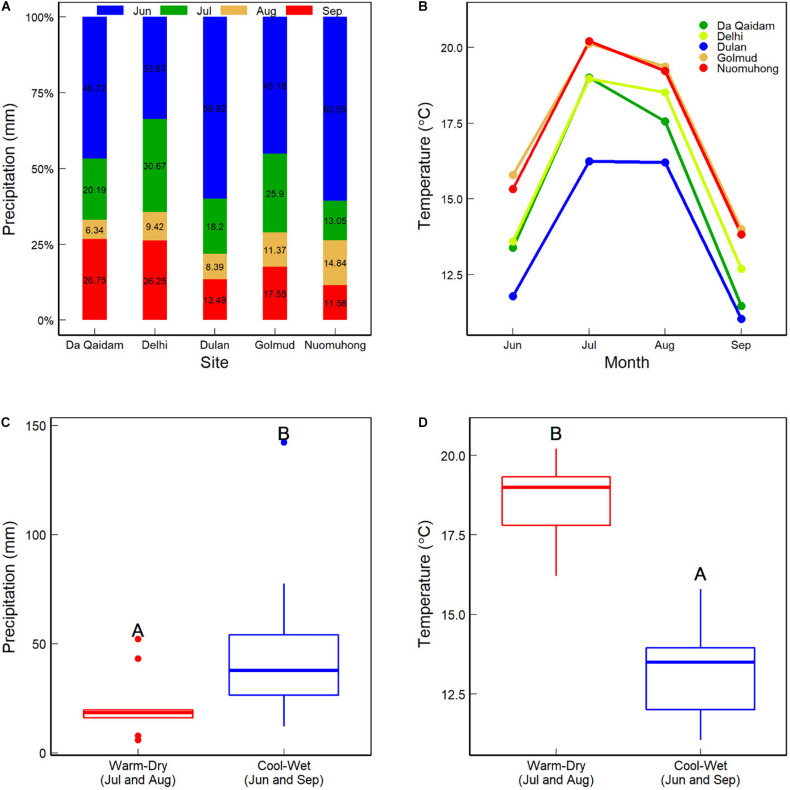
Precipitation **(A)** and air temperature **(B)** at each site from June to September during the plant growing season in 2010. **(C,D)** Show the sum precipitation and mean temperature between the warm-dry (July and August) and cool-wet (June and September) months.

There are 19 national meteorological stations within the Qaidam Basin. Daily records of temperature and precipitation of these stations were provided by the China Meteorological Data Service Center^1^ for 2005 (dry) and 2010 (wet). First, we integrated the daily temperature and precipitation into the plant growing season temperature (GST) and precipitation (GSP). The Kriging interpolation was used to produce climate rasters in ArcGIS10.2, and sit elevation was used as a covariate variable to improve the interpolation accuracy. The ratio of potential evapotranspiration (PET) to MAP describes the yearly aridity index. Finally, we extracted GST, GSP, and Aridity index values for each site according to its geographical coordinates. GSP and GST in 2010 were 37.5 mm more and 0.34°C higher than those in 2005, respectively (Table 1). The precipitation and temperature in June and July were 26.28 mm more and 5.25°C lower than in August and September 2010 (Figure 2). So, we defined July and August as warm-dry months and June and September as cool-wet ones for further analysis.

### Statistical Analyses

First, Two-way ANOVA was used to examine the effects of species identity and weather conditions on foliar δ^15^N and δ^13^C at the species level. Then, we examined the difference in CWMs of foliar δ^15^N and δ^13^C between 2005 and 2010, with the Kruskal-Wallis test by rank. In this step, we only considered the plant species sampled in both 2005 and 2010. Then, we examined the difference in the CWMs of foliar δ^15^N and δ^13^C between warm-dry and cool-wet months in 2010, with the Kruskal-Wallis test by rank. It is a non-parametric alternative to one-way analysis of variance (ANOVA) when the data does not meet the homogeneity assumptions of variance and normality.

Next, the CWMs of foliar δ^15^N and δ^13^C were treated as response variables while climate factors (GSP, GST, and Aridity), soil nutrients (SOC and STN) and physical properties (soil moisture, soluble soil salt, pH and bulk density) as potential predictors. Correlations between responsible and explanatory variables were examined. Bivariate regressions were used to examine how CWMs of foliar δ^13^C and δ^15^N vary along with each environmental variable. Finally, multivariate linear models were performed to investigate the main effect of climate and soil variables on the variance of foliar δ^15^N and δ^13^C at the community level. We followed a backward approach with Akaike Information Criterion (AIC) and Bayesian Information Criterion (BIC) to select the optimal models out (Rozenberg et al., 2011). The effect size (Eta squared, η^2^) was calculated as the proportion of the total variance explained by each factor in the most-fitted model.

All the analyses and visualizations were performed with R 4.0.2 (R Core Team, 2017).

## Results

### The Difference in δ^15^N and δ^13^C Between the Dry and Wet Years/Seasons

**FIGURE 3:**
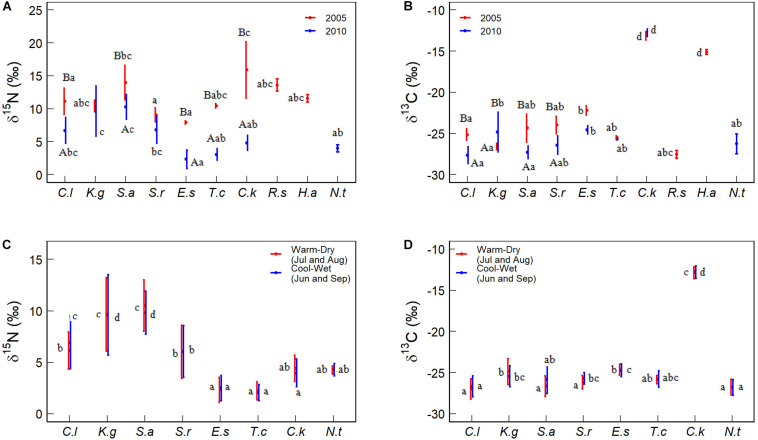
Comparison of foliar δ^15^N and δ^13^C among species. Different capital letters indicate significant differences between the two sampling years and between cool-wet and warm-dry months in 2010. Different lowercase letters indicate significant differences among species within a given year or months. The significance was at the *P* < 0.05 level. Species abbreviations were the same as Table 2.

Foliar δ^15^N and δ^13^C are different among species between 2005 and 2010. For a given species, foliar δ^15^N and δ^13^C were different between 2005 and 2010 and between cool-wet and warm-dry months in 2010. Specifically, the foliar δ^15^N of *Ceratoide latens* (11.12‰ vs 7.13‰), *Salsola abrotanoides* (13.97‰ vs 10.28‰), *Ephedra sinica* (7.91‰ vs 2.32‰), *Tamarix chinensis* (10.42‰ vs 3.06‰) and *Calligogum kozlovi* (15.90‰ vs 4.79‰) in 2005 were significantly higher than those in 2010 (Figure 3A). Foliar δ^15^N of *Kalidium gracile* (9.64‰) and *S. abrotanoides* (10.16‰) were higher than other species. Foliar δ^15^N of *E. sinica* (2.40‰) and *T. chinensis* (2.13‰) were lower than other species (5.58‰) between cool-wet and warm-dry months in 2010 (Figure 3C). Neither yearly (9.46 vs 8.11‰) nor monthly (6.52 vs 6.03‰) foliar δ^15^N was different between *C. latens* and *Sympegma regelii* (Figures 3A,C).

The foliar δ^13^C of *C. latens* (−25.14 vs −27.40‰), *S. abrotanoides* (−24.36 vs −27.32‰), and *S. regelii* (−23.99 vs −26.42‰) in 2005 were greatly higher than in 2010, but *K. gracile* (−26.60‰ vs −24.85‰) was different. The foliar δ^13^C of *C. kozlovi* (−12.85‰) and *H. ammodendron* (−15.11‰) were the highest (Figures 3B,D). The foliar δ^13^C of *E. sinica* was higher than *C. latens* at yearly (−23.78 vs −26.08‰) and monthly (−24.70 vs −26.85‰) scales. There was no evident difference in foliar δ^13^C for other species (Figures 3B,D).

**FIGURE 4:**
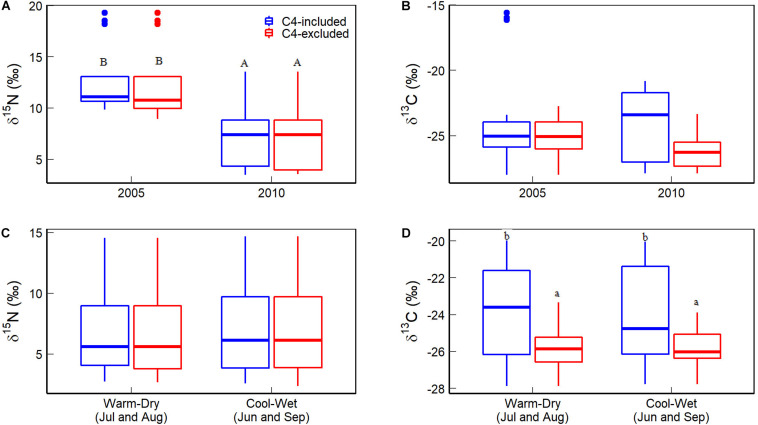
Comparisons of the community weighted means (CWMs) of foliar δ^15^N and δ^13^C. **(A,B)** Are for the difference between 2005 and 2010, while **(C,D)** are between cool-wet and warm-dry months in 2010). Different capital letters indicate a significant difference between different years or between cool-wet and warm-dry months. Different lowercase letters indicate a significant difference between C_4_ plants considered or not within a given year or months. The significance was given at the *P* < 0.05 level.

C_4_ plant (*C. kozlovi*) from two of the five sites had higher δ^15^N and cover in 2005 than in 2010 (15.90 vs 4.79‰ for foliar δ^15^N, and 2 vs 30–40% for cover, Table 2 and Figure 3A). Besides, the δ^15^N and cover values of other C_4_ plants were comparable to C_3_ ones. Consequently, the CWM of foliar δ^15^N in 2005 was around 12.56‰, approximately 5.3‰ higher than in 2010 (7.27‰), no matter C_4_ plants considered or not (Figure 4A).

The foliar δ^13^C of C_4_ plants, *C. kozlovi* (−13.13‰) and *H.ammodendron* (−15.11‰) in 2005 were much higher than the seven C_3_ plants (ranging from −29.19 to −21.84‰). The coverage of *C. kozlovi* (2%) was very low (Table 2 and Figure 3B), resulting in no difference in the CWM of foliar δ^13^C with and without C_4_ plants in 2005 (Figure 4B). However, the foliar δ^13^C of C_4_ plants, *C. kozlovi* (−12.76‰) was much higher than C_3_ plants (ranging from −29.19 to −22.63‰, Table 2 and Figure 3B) in 2010. No significant difference was found in the CWM of foliar δ^15^N between 2005 and 2010. However, the CWM of foliar δ^13^C in 2010 with C_4_ plants was 1.89‰ higher than that without C_4_ plants (Figure 4B).

The CWM of foliar δ^15^N in the cool-wet months (7.09‰) was slightly, only 0.21‰ higher than that in the warm-dry ones (6.88‰), but not significantly (*P* > 0.05, Figure 4C), the same for both C_3_ and C_4_ plants at the species level. The CWM of foliar δ^13^C with C_4_ plants (cool-wet: −23.95‰, warm-dry: −23.80‰) was significantly higher than without C_4_ ones (cool-wet: −25.76‰, warm-cool: −25.80‰) in 2010 (*P* > 0.05, Figure 3D).

### Foliar δ^15^N and δ^13^C Vary With Environmental Variables

**FIGURE 5:**
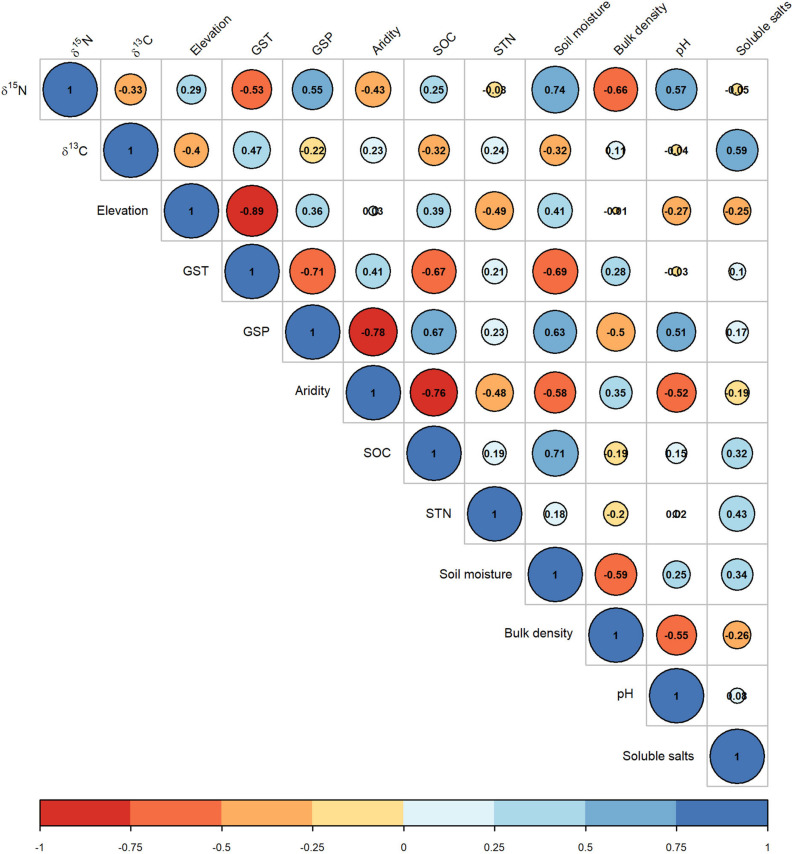
Correlation matrix of the community weighted means of foliar δ^15^N and δ^13^C with environmental variables. See abbreviations in Tables 1, 2.

The CWM of foliar δ^15^N was negatively correlated with δ^13^C (*r* = −0.33). The δ^15^N was closely linked with soil moisture, bulk density, pH, GSP, and GST, with their absolute coefficient values being higher than 0.5 (Figure 5). The CWM of foliar δ^13^C was closely correlated with soluble salt and GST, with absolute coefficients higher than 0.4. Meanwhile, the site aridity index was closely correlated with SOC and STN (Figure 5).

**FIGURE 6:**
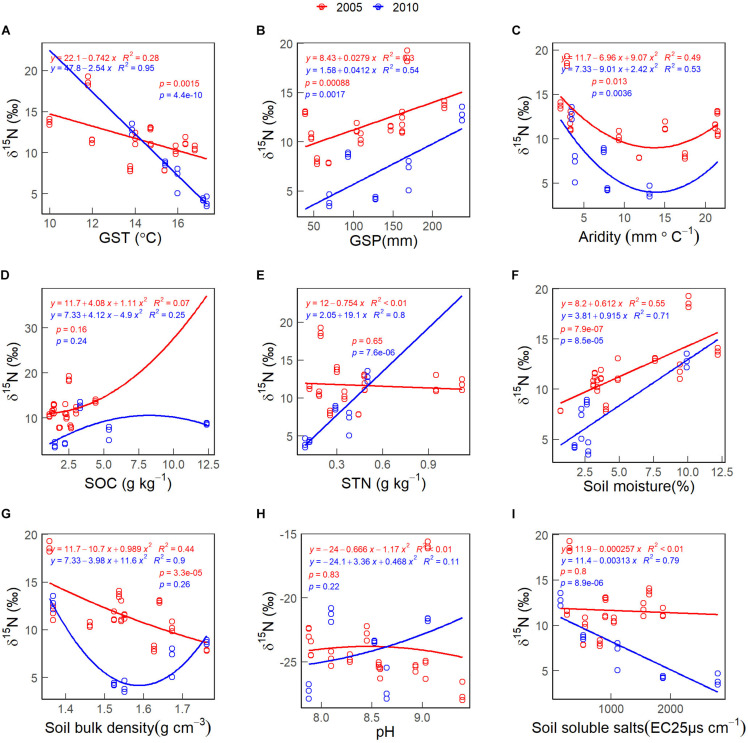
The patterns of the community weighted mean (CWM) of foliar δ^15^N along with environmental gradients, in 2005 (red lines & circles) and 2010 (blue lines & circles). **(A)** GST. **(B)** GSP. **(C)** Aridity. **(D)** SOC. **(E)** STN. **(F)** Soil moisture. **(G)** Soil bulk density. **(H)** pH. **(I)** Soil soluble salts. See abbreviations in Tables 1, 2.

The CWMs of foliar δ^15^N first decreased and increased with increasing habitat aridity index in 2005 and 2010 (Figure 6C). The CWM of foliar δ^15^N increases non-linearly with increasing soil pH values in 2005 (Figure 6H, red line). The CWM of foliar δ^15^N decreased with increasing GST (Figure 6A), soil bulk density in 2005 (Figure 6G, red line), and soil soluble salts in 2010 (Figure 6I, blue line). The CWM of foliar δ^15^N increased with increasing GSP (Figure 6B), STN in 2010 (Figure 6E, blue line) and soil moisture (Figure 6F). There was no significant correlation between the CWM of foliar δ^15^N and SOC (Figure 6D).

**FIGURE 7:**
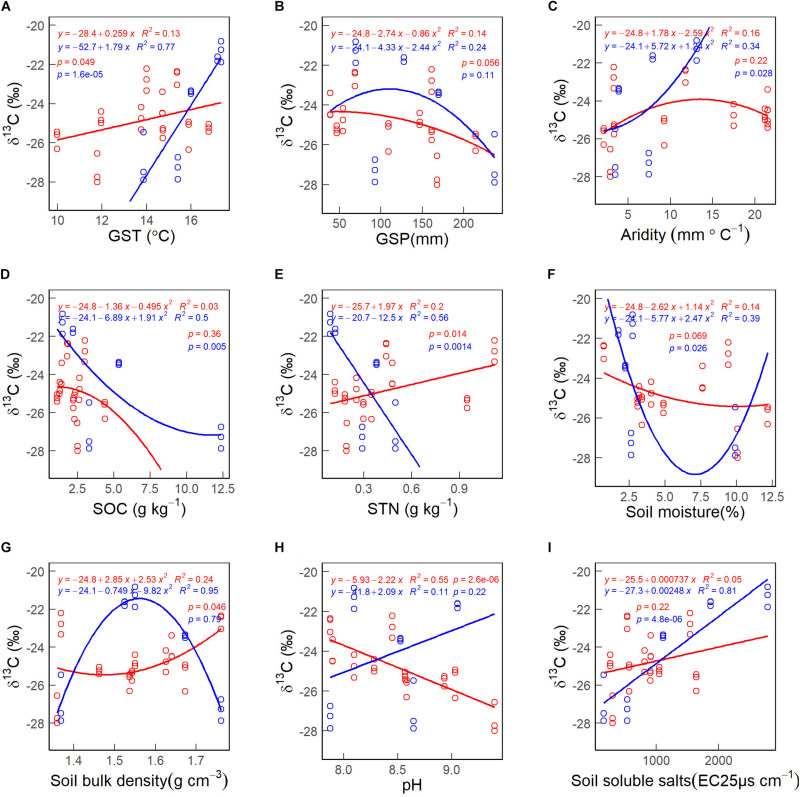
The patterns of the community weighted mean (CWM) of foliar δ^13^C along with environmental gradients, in 2005 (red lines & circles) and 2010 (blue lines & circles). **(A)** GST. **(B)** GSP. **(C)** Aridity. **(D)** SOC. **(E)** STN. **(F)** Soil moisture. **(G)** Soil bulk density. **(H)** pH. **(I)** Soil soluble salts. See abbreviations in Tables 1, 2.

There was no significant correlation between the CWM of foliar δ^13^C and GSP (Figure 7B). The CWMs of foliar δ^13^C first decreased and increased with increasing soil moisture in 2010 (Figure 7F). The CWM of foliar δ^13^C increases non-linearly with increasing soil bulk density values in 2005 (Figure 7G, red line) and habitat aridity index in 2010 (Figure 7C, blue line). The CWM of foliar δ^13^C decreases non-linearly with increasing SOC in 2010 (Figure 7D, blue line). The CWM of foliar δ^13^C decreased with increasing soil pH in 2005 (Figure 7H, red line) and STN in 2010 (Figure 7E, red line). The CWM of foliar δ^13^C increased with increasing GST (Figure 7A), STN in 2005 (Figure 7E, red line) and soil soluble salts in 2010 (Figure 7I, blue line).

Soil moisture alone explained 42% of the total variance of the CWM of foliar δ^15^N, followed by GST for 28% of the variance of the CWM of foliar δ^15^N within multivariate linear models (Table 3). Soluble soil salt content alone explained around 29.7% of the CWM of foliar δ^13^C, while GST explained 21.9% of the total variance of the CWM of foliar δ^13^C within the multivariate linear model (Table 3).

**TABLE 3:** Main effects of environmental variables on δ^15^N and δ^13^C in multivariate linear models.

	d.f.	SS	F	P	η^2^ (%)
**δ^15^N**					
GST	1	74.32	265.95	<0.01	28.04
GSP	1	16.58	59.34	<0.01	6.26
Aridity	1	1.04	3.72	0.07	0.39
STN	1	5.51	19.73	<0.01	2.08
SOC	1	47.05	168.38	<0.01	17.75
Soil moisture	1	111.63	399.47	<0.01	42.12
pH	1	1.92	6.86	<0.05	0.72
Residuals	25	6.99			
**δ^13^C**					
GST	1	60.84	13.54	<0.01	21.88
Soluble salt	1	82.47	18.36	<0.01	29.66
Residuals	30	134.78			

*d.f., the degree of freedom; SS, sum square, F, variance ratio; P, significance level; η^2^, Eta squared, the percentage of sum squares explained. See other abbreviations in Tables 1, 2.*

## Discussion

### Foliar δ^15^N and δ^13^C Vary Over Time

Foliar δ^15^N of desert plants is sensitive to climate dry-wet changes. At the species level, there was no significant difference in foliar δ^15^N range between C_3_ (1.29–18.82‰) and C_4_ plants (4.23–15.90‰) (Table 2). Apart from *K.gracile* (10.52 vs 9.66‰) and *S. regelii* (9.08 vs 6.81‰), foliar δ^15^N of other species in 2005 was notably higher than in 2010 (Figure 3A). There was no significant difference in foliar δ^15^N between cool-wet and warm-dry months for other species (Figure 3C). At the community level, the CWM of foliar δ^15^N in 2005 was higher than in 2010 (Figure 4A). The CWM of foliar δ^15^N in the cool-wet months was slightly higher than in the warm-dry ones, but not significantly (*P* > 0.05, Figure 4C).

These findings are consistent with previous relevant research that positive δ^15^N (6–10%) values are common in warm/arid regions (Lajtha and Schlesinger, 1986; Schulze et al., 1991), whereas low δ^15^N values (−4–0%) in cold/humid sites (Vitousek et al., 1989). Climate dry-wet change might affect soil nutrient availability. Amundson et al. (2003) reported that average soil δ^15^N followed similar patterns as foliar δ^15^N. The dry-wet changes significantly influence ^15^N retention and release from soils. Gaseous nitrogen losses are primarily responsible for large scale variational patterns of δ^15^N (Pataki et al., 2008; Bai and Houlton, 2009). Gaseous N loss (volatilization) would accelerate in dry climates and slowdown in wet ones to affect soil and foliar δ^15^N because ^14^N can more quickly release than ^15^N from the ground (Brenner et al., 2001).

Foliar δ^13^C of desert plants is mainly controlled by community assembly of C_3_ and C_4_ species. C_4_ plants discriminate less against ^13^C than C_3_ plants, and ^13^C are more enriched in C_4_ plans (Farquhar et al., 1982; Farquhar, 1983; Farquhar and Cernusak, 2012). This might be why the CWM of foliar δ^13^C in wetter conditions (2010) was higher than in drier ones (2005) when C_4_ plants were considered (Figure 4B). This finding is in line with Ghannoum et al. (2002) that C_4_ plants generally grow better in humid and warm habitats and that droughts can reduce foliar δ^13^C remarkably in most C_4_ grasses. Therefore, a higher foliar δ^13^C in a given community than the global average of C_3_ plants (about −27‰) can indicate the invasion or bloom of C_4_ plants and more humid conditions. In short, the higher CWM of foliar δ^13^C, the more C_4_ plants or, the more humid condition.

C_3_ plants are reported more enriched foliar δ^13^C in arid habitats where plants’ water-use strategies are more conservative than humid ones. Except for the foliar δ^13^C of *K. gracile* increased by 1.76‰, other four C_3_ plants declined their foliar δ^13^C in the wetter (2010) year (Figure 3B), compared to those in 2005 (Table 2 and Figure 3B). This finding suggests that *K. gracile* is less drought-tolerant than the other four species and prefers to live in a relatively humid environment. This might be why the difference in the CWMs of foliar δ^13^C was nonsignificant between the two sampling years when C_4_ plants were not considered (Figure 4B, red boxes).

However, there was no difference in the CWMs of foliar δ^13^C between warm-dry and cool-wet months (Figure 4D). It is because foliar δ^13^C of the seven C_3_ plants was similar between the warm-dry and cool-wet months in 2010 (Figure 3D). The result was consistent with Gatica et al. (2017) that foliar δ^13^C in three woody species did not increase toward sites with low precipitation or at the start of the plant growing season (the dry period). This phenomenon further indicates that climate change affects community δ^13^C via species assembly. As a valuable tool for long-term estimates of WUE (Farquhar et al., 1989), foliar δ^13^C does not respond significantly to short-time humidity change. The precipitation and temperature in the cool-wet season were 26.28 mm more and 5.25°C lower than in the wet-dry months in 2010 (Figure 2). In deserts, plants have developed stable drought tolerance. Therefore, minor differences in seasonal precipitation can not cause changes in the CWMs of foliar δ^13^C.

### Spatial Patterns of Foliar δ^15^N and δ^13^C

The water condition was the most crucial factor affecting plants nitrogen availability and controlling ecosystem N cycling. In our study, water condition (soil moisture and GSP) and GST were the vital factors influencing the CWM of foliar δ^15^N. On the one hand, water condition (soil moisture and GSP) explained 48.4% of the variance in the CWM of foliar δ^15^N (Table 3), followed by GST for 28% of the variance. The findings are partly consistent with Wu et al. (2019) that GSP was the most critical driver of δ^15^N variances in alpine grasslands on the northern Tibetan Plateau. Soil moisture can promote N mineralization and nitrification via microbial activity (Butterbach-Bahl and Gundersen, 2011; Chapin et al., 2011). On the other hand, edaphic factors (63%) explained the more significant variation in the CWM of foliar δ^15^N than climate factors (35%). The result coincides with Chapin et al. (2011) and Booth et al. (2005) that edaphic variables are critical in controlling ecosystem N cycling.

Ecosystem N losses increase with decreasing MAP and increasing MAT. That is because ^15^N-depleted gas can release more quickly from the ground (Amundson et al., 2003). We also found that the CWMs of foliar δ^15^N decreased fastly with increasing MAT (Figure 6A) and decreasing GSP (Figure 6B). The finding is consistent with Wu et al. (2019) but disagrees with Wang et al. (2014). Wang et al. (2014) found that foliar δ^15^N of grasses and shrubs remain stable with increasing aridity index. Such inconsistency may be due to the habitat conditions. Wang et al. (2014) conducted sampling in arid and semiarid grasslands, where the climate is not so dry as the Qaidam Basin. Besides, the species composition might be another reason why the foliar isotopic response to environmental changes differed. Specifically, Wang et al. (2014) analyzed grass and shrub genera while we only focused on dominant desert shrub species in this study.

Environmental factors also play an essential role in regulating the WUE of plant species. We found soluble soil salt content (29.7%) overrode GST (21.9%) to be the most critical driver for the CWMs of foliar δ^13^C (Table 3). The finding was consistent with Yang et al. (2015) that edaphic rather than climatic variables were better predictors of ^13^C enrichment at high altitudes. In this study, the CWMs of foliar δ^13^C had positively correlated with soil soluble salts and GST (Figures 7A,I), to some extent being consistent with previous studies (Brugnoli and Lauteri, 1991; Wang and Schjoerring, 2012; Wu et al., 2013; Loader and Hemming, 2016; Min et al., 2017).

In Golmud-4, GST and soil soluble salts were 1.75°C and 1870 EC25 μs cm^–1^ higher, GSP was 87.58 mm less, and the CWM of foliar δ^13^C (−21.32‰) was 3.52‰ higher than other four sites (−24.84‰) in 2010 (Tables 1, 2 and Figure 7). It could be because the higher soil salt content increases soil solution’s osmotic pressure, reduces soil water potential, and changes soil’s physical and chemical properties (Khasa et al., 2002). The increased salt stress is likely to induce stomatal closure, decrease the partial pressure of ^12^CO_2_, force stomatal to absorb more ^13^CO_2_, and finally increase δ^13^C value in plants (del Amor, 2013). The possible explanation for the positive δ^13^C-GST correlation is that water vapor pressure increases with temperature, making soil moisture and plant transpiration enhanced. Under arid conditions, plants’ stomatal conductance will decrease, resulting in decreased C_*i*_/C_*a*_ value and an increase of δ^13^C when soil available moisture reduces (Morecroft and Woodward, 1996).

## Conclusion

This study examined the differences in δ^15^N and δ^13^C of desert plants under climate change and disentangled climate and edaphic factors’ relative contribution to the variance in δ^15^N and δ^13^C. We further analyzed the interspecific variation in δ^15^N and δ^13^C under the weather dry-wet changes. First, foliar δ^15^N and δ^13^C was higher in relatively dry conditions compared to wet conditions. Second, foliar δ^15^N and δ^13^C primarily affected by soil factors, followed by temperature. Soil moisture was the most critical driver for foliar δ^15^N, which explained 42.1% of the variance alone. However, the total soluble salt content was the crucial factor in foliar δ^13^C, responsible for 28.7% of the variance. GST explained 28.0 and 21.9% of the variance in foliar δ^15^N and δ^13^C of desert plants in the Qaidam Basin. Besides, foliar δ^15^N and δ^13^C are also affected mainly by inter-species differences. In the future, plant functional diversity, like CWMs, can examine how desert species respond to climate change and human disturbance.

## Data Availability Statement

The original contributions presented in the study are included in the article/Supplementary Material, further inquiries can be directed to the corresponding author/s.

## Author Contributions

HC and JW designed the study. HC conducted field surveys. JW led the writing. WN analyzed the data and wrote the first draft under JW’s help. JW and HC revised the text thoroughly and interpreted the results. All authors contributed to this work and approved the final manuscript before submission.

## Conflict of Interest

The authors declare that the research was conducted in the absence of any commercial or financial relationships that could be construed as a potential conflict of interest.

## Footnotes

1 http://data.cma.cn/en

## Supplementary Material

Supplementary Table 1 Site information of δ^15^N and δ^13^C during the growing season in 2010 in the Qaidam Basin, northeastern Qinghai-Tibetan Plateau.

Supplementary Table 2 The full models of multivariate linear for δ^15^N and δ^13^C. d.f., the degree of freedom; SS, sum square, F, variance ratio; P, significance level; η2, Eta squared, the percentage of sum squares explained. See other abbreviations in Tables 1, 2.
